# Research on the application of tree drawing projection tests in the condition assessment of depression

**DOI:** 10.3389/fpsyt.2026.1794378

**Published:** 2026-05-22

**Authors:** Guorui Liu, Jin Wang, Guanxiong Li, Tingyu Liu, Yanfei Zhang

**Affiliations:** 1 Department of Medical Psychology, Second Affiliated Hospital of Naval Medical University, Shanghai, China; 2 Department of Medical Psychology, No. 905 Hospital of PLA Navy, Shanghai, China; 3 Suzhou Guangji Hospital, Suzhou, China; 4 Department of Medical Psychology, The 908th Hospital of PLA Joint Logistic Support Force, Nanchang, China; 5 Department of Laboratory Medicine, Second Affiliated Hospital of Naval Medical University, Shanghai, China

**Keywords:** condition assessment, depression, projection tests, quantitative research, tree drawing projection

## Abstract

**Objective:**

This study aims to extract and quantitatively analyze the tree drawing projection indices from groups of patients with depression, patients in remission, and a normal control group, to explore characteristic indicators of tree drawing projections in individuals with depression and provide a potential basis for condition assessment of depression.

**Methods:**

Tree drawing tests were administered to 118 patients with depression, 114 patients in remission, and 118 normal controls. Computer image recognition and data collection were used for quantitative analysis of the tree projections, and statistical analysis was conducted on the results from the three groups.

**Results:**

ANOVA tests revealed significant statistical differences between the depression patients, remission patients, and normal controls in the following quantitative indices: canopy area, canopy height, canopy width, trunk area, trunk width, total area, and the ratio of canopy width to trunk width (*p* values: < 0.001, < 0.001, < 0.001, 0.003, < 0.001, 0.004, < 0.001). No significant differences were found in trunk height, root width, root height, root area, total height, the ratio of canopy height to trunk height, and the ratio of canopy area to trunk area. Further LSD-t and Tukey HSD tests showed that compared to the depression group, the remission group exhibited significant differences in canopy area, canopy height, canopy width, trunk area, trunk width, the ratio of canopy width to trunk width, and total area (*p* values: 0.001, < 0.001, 0.009, 0.002, < 0.001, 0.007, < 0.001). No significant differences were found in the other indices; also, no significant differences were found between the remission group and the normal control group across all 14 indices.

**Conclusion:**

There are seven quantitative indices where significant statistical differences exist among the tree drawings from the depression group, the remission group, and the normal control group, and seven indices where no significant differences were found. In this study, the quantitative indices of tree drawing projections have value in differentiating between the depression group, the remission group, and the healthy control group.

## Introduction

Depressive symptoms are clinical manifestations of mood disorders, characterized by persistently low mood, slowed thinking, cognitive impairment, reduced volitional activity, and physical symptoms (1, 2). Depression, a type of mood disorder, is marked by these clinical features and leads to impaired social functioning, decreased work efficiency, and even self-harm or suicide. It is recurrent and poses a substantial economic burden on individuals, families, and society (1). Research indicates that depression has become a major contributor to the global burden of disease, with increasing incidence rates in recent years. However, the typical treatment duration for depression exceeds six weeks, emphasizing the need for timely diagnosis and intervention to prevent the progression of symptoms (3, 4). Despite the increasing detail in diagnostic manuals such as the DSM and ICD, challenges remain in diagnosing depression, including the subjectivity of diagnostic criteria and the low diagnosis rate in primary care settings. Traditional diagnostic methods often rely on interviews and self-report questionnaires, which may lack sufficient objectivity and accuracy. Consequently, researchers are seeking new diagnostic tools to enhance diagnostic accuracy and objectivity.

In this context, the search for more objective and effective diagnostic tools has become a research focus in the field of mental health. The Tree Drawing Projection Test, a psychological projective test, has proven useful in assessing depression and other emotional disorders. The test relies on the free expression of subjects to non-specific stimuli, revealing their inner world and emotional state through the interpretation of tree drawings (5–8). Compared to traditional tests, the Tree Drawing Projection Test is easier to administer, imposes less psychological stress, and can reduce intentional control by subjects (6, 9–11). Researchers domestically and internationally have found the Tree Drawing Projection Test valuable in the auxiliary diagnosis of diseases such as schizophrenia, depression, somatization symptoms, anxiety disorders, and Alzheimer’s disease (5, 6, 9, 12–16). However, most studies on tree drawing projection remain at the subjective qualitative research level, lacking quantitative data collection and indicator analysis (5).

Our team developed Tree Drawing Projection Test software in 2017, which, based on the data collection of tree drawing length, width, and height, employs computer image recognition technology to scan and collect data on the area of the tree crown, trunk, and roots in tree projections, thus enhancing the objectivity and accuracy of diagnosis through quantitative analysis of indicators. Previous research has identified differences in tree drawings between patients with depression and healthy individuals (14). This comparative study examines changes in Tree Drawing Projection Test indicators before and after symptom relief in depression patients, and the differences in indicators between post-relief patients and healthy individuals, further exploring the value of the Tree Drawing Projection Test in the condition assessment of depression.

## Methods

This study was conducted at the Suzhou Mental Health Center in Suzhou, China. Participants included a depression case group recruited from the inpatient wards and a normal control group recruited from the community. The study received approval from the Medical Ethics Committee of Suzhou Guangji Hospital (Approval No.: 2021-012). All subjects were informed in detail about the purpose and procedures of the study and provided written consent before participation. Recruitment occurred from February 2021 to June 2022.

### Recruitment criteria

The depression case group met the DSM-5 diagnostic criteria for depression (17), with participants aged between 18 and 60 years, regardless of gender, and a HDRS-21 score of ≥21 (18). The depression remission group comprised discharged patients who, upon admission, met the DSM-5 diagnostic criteria for depression (17) and had a HDRS-21 score of ≥21 (18). At discharge, these patients had a HDRS-21 score reduction rate of over 50%, indicating an alleviation of depressive symptoms. The normal control group consisted of individuals from the same region, recruited during the same period (2021-2022). Inclusion criteria for the control group were the absence of significant psychiatric symptoms (no positive factors on the SCL-90) and no history of mental illness, with gender and age matching the case group.

In this study, patients with depression were treated with antidepressant medications, including escitalopram, sertraline, venlafaxine, and other similar antidepressants. The duration of inpatient treatment ranged from 4 to 6 weeks. Additionally, no comorbid psychiatric conditions were present in the patient group.

### Measures

#### Tree drawing projection test

Each participant was provided with A4 printing paper and a black or blue-black pen. Participants were instructed to draw according to the following guidelines:

The drawing projection test is not a test of drawing skills, and the drawing does not need to be aesthetically pleasing.

The drawing projection test is not a still life drawing, and it does not need to resemble real-life objects.

If you cannot draw what you intend to, you may draw a circle and write a Chinese character inside it as a substitute.

Before drawing the tree, close your eyes and meditate for half a minute.

Draw the tree that appears in your meditation. If no tree appears, open your eyes and draw the tree you most want to draw.

After completing your drawing, write your age and gender on the drawing paper.

The testing conditions were standardized across all participants, with a fixed 5-minute time limit for the drawing task. All participants received identical instructions to ensure consistency across the study.

### Instruments

#### Epson high-definition scanner (DS-1630)

This study utilized an Epson High-Definition Scanner (DS-1630) to digitize tree projection drawings. The scanned images were stored on a computer.

#### Image data scanning and collection software

**Figure 1:**
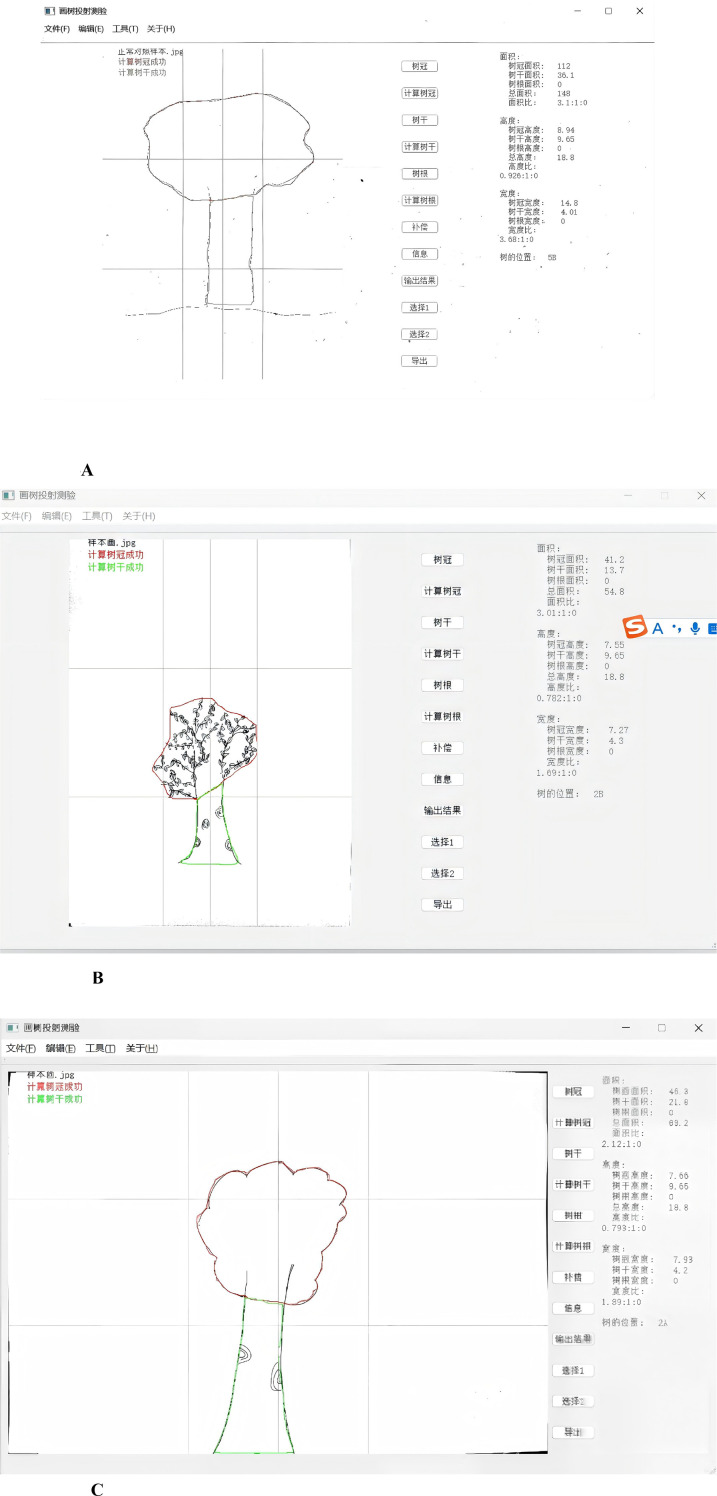
**(A)** The tree drawing projection test software calculated various metrics of the normal control group. **(B)** The tree drawing projection test software calculated various metrics of the depression group. **(C)** The tree drawing projection test software calculated various metrics of the depression remission group.

The research employed the Tree Drawing Projection Test software, a custom-developed tool designed to extract quantitative features from tree drawings, such as crown area, trunk area, height, width, and length. The software operates by allowing users to trace the contours of the tree drawings using a mouse, after which it automatically computes these structural metrics in centimeters, based on the contours traced (**Figure 1**). To ensure measurement reliability, the data were processed using a standardized scanning procedure involving a fixed-resolution scanning device and consistent scanning settings. All image processing followed a uniform protocol across all sessions.

The reliability and validity of the software have been assessed through several steps:

Reliability: Test-retest reliability and inter-rater reliability were assessed in previous studies. Test-retest reliability was evaluated by comparing the software’s measurements at different time points for the same set of tree drawings. Inter-rater reliability was measured by having multiple users trace and measure the same set of drawings, with results showing a high degree of consistency across raters.

Validity: The validity of the software was assessed through comparisons with clinical evaluations. Clinical evaluations indicated that the software was able to effectively differentiate between patients with depression and healthy controls, supporting its construct validity (12, 19). This suggests that the software is capable of capturing meaningful differences between clinical groups.

Calibration and Standardization Procedures: Calibration of the software was conducted using a set of reference drawings, ensuring that measurements were accurate and consistent across different systems and operators. Standardization procedures were applied to the scanning and image processing steps to reduce variability and ensure uniformity.

The software has been validated in previous studies, demonstrating its capacity to accurately measure and differentiate tree drawing features, showing strong reliability and validity for clinical use.

### Statistical methods

Statistical analyses were performed using SPSS version 26.0. ANOVA with *post-hoc* LSD and Tukey HSD tests were used for group comparisons. Assumption tests for normality (Shapiro-Wilk test) and homogeneity of variance (Levene’s test) were conducted. Effect sizes were calculated using Cohen’s d. A power analysis, performed with G*Power software, estimated the required sample size for detecting significant differences with 80% power at an alpha level of 0.05, assuming a medium effect size (Cohen’s d = 0.5). The analysis indicated that at least 118 participants per group were needed. In the present study, 118 participants were included in the depression group, 114 in the depression remission group, and 118 in the normal control group, indicating that the sample size was generally sufficient to achieve the desired statistical power. A p-value of <0.05 was considered statistically significant.

## Results

### Basic demographic characteristics

This study included a group of 118 patients diagnosed with depression, aged between 18 and 60 years, comprising 40 males and 78 females. The depression remission group consisted of 114 individuals, including 44 males and 70 females. The normal control group included 118 subjects, aged between 18 and 60 years, with 50 males and 68 females. **Table 1** presents the demographic data of the sample. The distribution of age and gender was similar across the groups, and no statistically significant differences were observed (*p* > 0.05).

**Table 1:** Comparison of demographic differences across groups.

Variable	Depression group	Remission group	Normal control group	*F*	*p*
Age	41.10 ± 12.068	38.53 ± 10.202	36.95 ± 10.474	1.222	0.297
Sex				0.900	0.638
Male	40	44	50		
Female	78	70	68		

### Comparison among depression, depression remission, and normal control groups

Prior to conducting the ANOVA, normality tests were performed on the data for each group using the Shapiro-Wilk test. The results indicated that the data in all groups followed a normal distribution (*p* > 0.05). Additionally, Levene’s test for homogeneity of variance revealed no significant differences in variance across the groups (*p* > 0.05), confirming that the assumption of homogeneity of variance was met.

ANOVA was then conducted using the Tree Drawing Projection Test software for data acquisition and statistical analysis. Significant differences were found among the three groups in the following quantitative metrics: canopy area, canopy height, canopy width, trunk area, trunk width, total area, and the ratio of canopy width to trunk width (*p* values: < 0.001, < 0.001, < 0.001, 0.003, < 0.001, 0.004, < 0.001, respectively). No significant differences were observed in trunk height, root width, root height, root area, total height, the ratio of canopy height to trunk height, and the ratio of canopy area to trunk area (see **Table 2**).

**Table 2:** Comparison of tree drawing indicators among depression, depression remission, and normal control groups.

Variable	Depression group (mean ± SD, 95% CI)	Remission group (mean ± SD, 95% CI)	Normal control group (mean ± SD, 95% CI)	*F*	*p*	Cohen’s d (depression vs. remission)	Cohen’s d (depression vs. normal control)	Cohen’s d (remission vs. normal control)
Canopy area(cm2)	46.87 ± 13.01 (44.52, 49.22)	83.38 ± 25.03 (78.79, 87.97)	90.84 ± 25.95 (86.16, 95.52)	10.546	**< 0.001**	-1.901	-2.097	-0.293
Canopy height(cm)	6.47 ± 1.62 (6.18, 6.76)	9.69 ± 2.74 (9.19, 10.19)	10.33 ± 2.85 (9.82, 10.84)	13.575	**< 0.001**	-1.911	-2.284	-0.382
Canopy width(cm)	7.90 ± 1.98 (7.54, 8.26)	9.98 ± 2.48 (9.52, 10.44)	10.50 ± 2.91 (9.97, 11.03)	6.119	**0.003**	-0.924	-0.999	-0.186
Trunk area(cm2)	8.73 ± 2.30 (8.32, 9.14)	16.47 ± 4.64 (15.62, 17.32)	19.38 ± 5.39 (18.41, 20.35)	10.095	**< 0.001**	-2.105	-2.379	-0.581
Trunk height(cm)	4.97 ± 0.30 (4.92, 5.02)	4.77 ± 0.33 (4.71, 4.83)	5.07 ± 0.34 (5.01, 5.13)	0.125	0.882	0.642	-0.322	-0.968
Trunk width(cm)	1.76 ± 0.32 (1.70, 1.82)	4.49 ± 1.30 (4.25, 4.73)	5.09 ± 1.69 (4.79, 5.39)	37.194	**< 0.001**	-2.564	-2.978	-0.410
Root area(cm2)	1.97 ± 0.46 (1.89, 2.05)	3.81 ± 1.62 (3.51, 4.11)	4.38 ± 1.35 (4.14, 4.62)	0.804	0.449	-1.426	-2.153	-0.383
Root height(cm)	0.42 ± 0.09 (0.40, 0.44)	1.30 ± 0.36 (1.23, 1.37)	1.46 ± 0.38 (1.39, 1.53)	1.963	0.144	-2.749	-3.252	-0.501
Root width(cm)	1.03 ± 0.26 (0.98, 1.08)	1.12 ± 0.33 (1.06, 1.18)	1.23 ± 0.34 (1.17, 1.29)	0.059	0.943	-0.302	-0.671	-0.372
Ratio of canopy area to trunk area	40.42 ± 19.17 (36.96, 43.88)	10.47 ± 4.78 (9.59, 11.35)	9.04 ± 4.34 (8.26, 9.82)	2.587	0.078	-1.271	-1.500	-0.352
Ratio of canopy height to trunk height	2.21 ± 0.48 (2.12, 2.30)	3.29 ± 0.54 (3.19, 3.39)	3.30 ± 0.69 (3.18, 3.42)	2.734	0.068	-1.193	-1.324	-0.289
Ratio of canopy width to trunk width	11.44 ± 0.33 (11.38, 11.50)	4.39 ± 1.73 (4.07, 4.71)	3.66 ± 0.83(3.51, 3.81)	5.617	**0.004**	-2.388	-3.226	-0.439
Total area(cm2)	57.62 ± 17.69 (54.43, 60.81)	103.58 ± 24.69 (99.05, 108.11)	114.53 ± 23.81 (110.23, 118.83)	12.241	**< 0.001**	-1.357	-1.526	-0.264
Total height(cm)	11.81 ± 3.20 (11.23, 12.39)	13.53 ± 3.53 (11.85, 15.21)	14.39 ± 4.10 (12.88, 14.18)	2.827	0.062	-0.466	-0.768	-0.374

All values are presented as mean ± SD (95% CI).

Cohen’s d values indicate the effect sizes for pairwise comparisons between groups.

Significant p-values (p < 0.05) are bolded.

Effect sizes and p-values for pairwise comparisons were calculated based on the results from the one-way ANOVA.

### Comparison between the depression group and the normal control group

Following ANOVA, further analysis was conducted using both LSD and Tukey HSD *post-hoc* tests. It was found that, compared to the normal control group, the trees drawn by patients in the depression group showed significant differences in canopy area, canopy height, canopy width, trunk area, trunk width, the ratio of canopy width to trunk width, and total area (*p* values respectively: LSD: < 0.001, < 0.001, 0.001, < 0.001, < 0.001, 0.003, < 0.001; Tukey HSD: < 0.001, < 0.001, 0.003, < 0.001, < 0.001, 0.008, < 0.001). However, for total height, while LSD showed a significant difference (*p* = 0.021), Tukey HSD did not reach statistical significance (*p* = 0.053), indicating a borderline result (see **Table 3**). No significant differences were observed in trunk height, root area, root height, root width, the ratio of canopy area to trunk area, and the ratio of canopy height to trunk height according to both LSD and Tukey HSD tests (*p* values: LSD: 0.862, 0.226, 0.067, 0.732, 0.045, 0.043; Tukey HSD: 0.983, 0.446, 0.159, 0.937, 0.111, 0.107) (see **Table 3**).

**Table 3:** Comparison of tree drawing indices between the depression group and the normal control group.

Variable	Depression group	Normal control group	*p (LST)*	*p* (HSD)
Canopy area(cm^2^)	46.87 ± 13.01	90.84 ± 25.95	< 0.001	< 0.001
Canopy height(cm)	6.47 ± 1.62	10.33 ± 2.85	< 0.001	< 0.001
Canopy width(cm)	7.90 ± 1.98	10.50 ± 2.91	0.001	0.003
Trunk area(cm^2^)	8.73 ± 2.30	19.38 ± 5.39	< 0.001	< 0.001
Trunk height(cm)	4.97 ± 0.30	5.07 ± 0.34	0.862	0.983
Trunk width(cm)	1.76 ± 0.32	5.09 ± 1.69	< 0.001	< 0.001
Root area(cm^2^)	1.97 ± 0.46	4.38 ± 1.35	0.226	0.446
Root height(cm)	0.42 ± 0.09	1.46 ± 0.38	0.067	0.159
Root width(cm)	1.03 ± 0.26	1.23 ± 0.34	0.732	0.937
Ratio of canopy area to trunk area	40.42 ± 19.17	9.04 ± 4.34	0.045	0.111
Ratio of canopy height to trunk height	2.21 ± 0.48	3.30 ± 0.69	0.043	0.107
Ratio of canopy width to trunk width	11.44 ± 0.33	3.66 ± 0.83	0.003	0.008
Total area(cm^2^)	57.62 ± 17.69	114.53 ± 23.81	< 0.001	< 0.001
Total height(cm)	11.81 ± 3.20	14.39 ± 4.10	0.021	0.053

### Comparison between the depression group and the depression remission group

Compared to the depression remission group, significant differences were observed in the trees drawn by the depression group in terms of canopy area, canopy height, canopy width, trunk area, trunk width, the ratio of canopy width to trunk width, and total area (*p* values respectively: LSD: 0.001, < 0.001, 0.009, 0.002, < 0.001, 0.007, < 0.001; Tukey HSD: 0.001, < 0.001, 0.026, 0.006, < 0.001, 0.019, 0.001). However, for the ratio of canopy height to trunk height, while LSD showed a significant difference (*p* = 0.046), Tukey HSD did not reach statistical significance (*p* = 0.113), indicating a borderline result (see **Table 4**). No significant differences were found in trunk height, root area, root height, root width, the ratio of canopy area to trunk area, or total height (*p* values: LSD: 0.748, 0.359, 0.124, 0.876, 0.058, 0.126; Tukey HSD: 0.945, 0.628, 0.271, 0.987, 0.139, 0.275) (see **Table 4**).

**Table 4:** Comparison of tree drawing indices between the depression group and the depression remission group.

Variable	Depression group	Remission group	*p (LST)*	*p* (HSD)
Canopy area(cm^2^)	46.87 ± 13.01	83.38 ± 25.00	0.001	0.001
Canopy height(cm)	6.47 ± 1.62	9.69 ± 2.74	< 0.001	< 0.001
Canopy width(cm)	7.90 ± 1.98	9.98 ± 2.48	0.009	0.026
Trunk area(cm^2^)	8.73 ± 2.30	16.47 ± 4.64	0.002	0.006
Trunk height(cm)	4.97 ± 0.30	4.77 ± 0.33	0.748	0.945
Trunk width(cm)	1.76 ± 0.32	4.49 ± 1.30	< 0.001	< 0.001
Root area(cm^2^)	1.97 ± 0.46	3.81 ± 1.62	0.359	0.628
Root height(cm)	0.42 ± 0.09	1.30 ± 0.36	0.124	0.271
Root width(cm)	1.03 ± 0.26	1.12 ± 0.33	0.876	0.987
Ratio of canopy area to trunk area	40.42 ± 19.17	10.47 ± 4.78	0.058	0.139
Ratio of canopy height to trunk height	2.21 ± 0.48	3.29 ± 0.54	0.046	0.113
Ratio of canopy width to trunk width	11.44 ± 0.33	4.39 ± 1.73	0.007	0.019
Total area(cm^2^)	57.62 ± 17.69	103.58 ± 24.69	< 0.001	0.001
Total height(cm)	11.81 ± 3.20	13.53 ± 3.53	0.126	0.275

### Comparison between the depression remission group and the normal control group

Compared to the normal control group, no significant differences were found in the trees drawn by the depression remission group regarding canopy area, canopy height, canopy width, trunk area, trunk height, trunk width, root area, root height, root width, the ratio of canopy area to trunk area, the ratio of canopy height to trunk height, the ratio of canopy width to trunk width, total area, and total height (see **Table 5**).

**Table 5:** Comparison of tree drawing indices between the depression remission group and the normal control group.

Variable	Remission group	Normal control group	*p (LST)*	*p* (HSD)
**Canopy area(cm^2^)**	83.38 ± 25.00	90.84 ± 25.95	0.471	0.751
**Canopy height(cm)**	9.69 ± 2.74	10.33 ± 2.85	0.421	0.705
**Canopy width(cm)**	9.98 ± 2.48	10.50 ± 2.91	0.513	0.316
**Trunk area(cm^2^)**	16.47 ± 4.64	19.38 ± 5.39	0.241	0.467
**Trunk height(cm)**	4.77 ± 0.33	5.07 ± 0.34	0.862	0.874
**Trunk width(cm)**	4.49 ± 1.30	5.09 ± 1.69	0.148	0.316
**Root area(cm^2^)**	3.81 ± 1.62	4.38 ± 1.35	0.777	0.957
**Root height(cm)**	1.30 ± 0.36	1.46 ± 0.38	0.782	0.958
**Root width(cm)**	1.12 ± 0.33	1.23 ± 0.34	0.855	0.982
**Ratio of canopy area to trunk area**	10.47 ± 4.78	9.04 ± 4.34	0.927	0.995
**Ratio of canopy height to trunk height**	3.29 ± 0.54	3.30 ± 0.69	0.991	0.995
**Ratio of canopy width to trunk width**	4.39 ± 1.73	3.66 ± 0.83	0.771	0.957
**Total area(cm^2^)**	103.58 ± 24.69	114.53 ± 23.81	0.374	0.647
**Total height(cm)**	13.53 ± 3.53	14.39 ± 4.10	0.438	0.717

## Discussion

Currently, the clinical diagnosis and severity assessment of depression primarily rely on clinician interviews combined with patient history and supplemented by scale assessments. Despite the diagnostic standards provided by manuals such as the DSM and ICD, these methods cannot completely avoid the drawback of subjectivity in assessments (5). Projection tests, as one of the three major techniques in psychology, can compensate for the deficiencies of scales. Existing studies have confirmed the correlation between tree drawing projection and depression. However, tree drawing projection tests have their limitations, such as non-standard scoring and interpretation, lack of consistency, and subjectivity in the selection of drawing features by researchers, making it difficult to compare results across different studies (20, 21). Moreover, interpretations of certain drawing features vary; for instance, some researchers consider drawing a “chimney” as a negative expression of family or internal conflicts, while others view it as a negative expression in general (22).

In this study, our team utilized modern imaging scanning and computer image recognition technologies to conduct quantitative analysis of metrics such as canopy size and shape, trunk thickness, etc., thereby enhancing the objectivity and accuracy of diagnostics. Early results have preliminarily validated the value of tree drawing projections in assisting the diagnosis of depression (12, 14). This comparative study of tree drawing indices among the depression, depression remission, and normal control groups revealed statistically significant differences in canopy area, canopy height, canopy width, trunk area, trunk width, total area, and the ratio of canopy width to trunk width (*p* < 0.05), while no significant differences were found in trunk height, root width, root height, root area, total height, the ratio of canopy height to trunk height, and the ratio of canopy area to trunk area (see **Table 2**). These findings affirm the value of tree drawing projection tests in differentiating between different groups. Further, the study conducted LSD-t tests to explore the application value of tree drawing indices in the progression of depression symptoms.

Previous studies have indicated that the canopy in tree drawings primarily reflects a person’s thinking, cognitive abilities, and social interactions (15, 23). Comparisons between the depression group, the depression remission group, and the normal control group (see **Tables 3**, **4**) revealed that the drawn canopy width, height, and area were smaller (*p* < 0.05). This corresponds to issues in these areas and aligns with symptoms reported by patients with depression, such as slowed thinking, helplessness, lack of interest, diminished capability, poor concentration, cognitive decline, and reluctance to go out and socialize. While these findings are consistent with previous research results, it is important to note that the specific relationship between canopy size and cognitive or emotional states should be interpreted with caution, as further empirical evidence is needed to support these direct associations. The overall tree mainly represents the current psychological and mental state of the drawer; smaller total tree areas in the depression group compared to the remission and control groups suggest poor psychological states, low energy, or feelings of inferiority and lack of self-confidence (19, 24).

In tree drawing projection tests, the trunk primarily reflects the emotional state of the drawer (25). A smaller trunk area in the depression group compared to the depression remission group and the normal control group (*p* < 0.05) may indicates emotional issues (see **Tables 2**, **3**), characterized by unhappiness, low mood, and negativity. Scholars such as Ji Yuanhong believe that in tree drawing tests, the width of the tree can reflect the emotional stability of the drawer (26, 27). A wider trunk indicates more stable emotions. LSD-t test results showed that the trunk width was narrower in the depression group compared to the remission and control groups, indicating characteristics of emotional fragility and instability. However, these interpretations should be viewed as suggestive rather than conclusive, and further validation studies are necessary to confirm the emotional implications of trunk size in the context of depression.

In tree drawing projection tests, the canopy, trunk, and roots represent the superego, ego, and id of the drawer, respectively (26). In this study, the ANOVA and LSD-t results for the three groups showed no statistical differences in root area, width, and height. The roots represent an individual’s instincts and subconscious. Some scholars also believe that roots reflect the collective subconscious of the drawer’s community (25), that is, the customs or habits formed by the long-term living conditions of their region and community. The lack of significant differences in the three root indicators in this study might be tentatively attributed to the influence of Confucian and other cultural norms prevalent in Chinese society, which advocate restraint and moderation towards instinctual behaviors. However, this interpretation is speculative and should be considered with caution, as it is not directly supported by the data presented. Further research is needed to explore the potential cultural influences on tree projection test drawings.

The ANOVA results for total height showed no significant differences across the three groups, and the LSD-t test results for the depression and depression remission groups also indicated no significant differences in trunk height and total height. This is consistent with previous research, suggesting that compared to area and width, height is not a sensitive indicator of changes in depressive symptoms (19).

The results of this study indicate that there are no statistically significant differences between the depression remission group and the normal control group across various indices, including canopy area, canopy height, canopy width, trunk area, trunk height, trunk width, root area, root height, root width, the ratio of canopy area to trunk area, the ratio of canopy height to trunk height, the ratio of canopy width to trunk width, total area, and total height. This suggests that as the symptoms of depression improve—such as slowed thinking, reduced volition and behavior, and low mood—the values for tree canopy area, height, width, trunk area, width, total area, and total height tend to increase. Although the statistical analysis shows no significant differences between the depression remission group and the normal control group (see **Table 5**), the average values for canopy area, height, width, trunk area, width, total area, and total height in the depression remission group are still less than those in the normal control group, indicating that cognitive, social interaction, and other functions have not fully returned to normal levels immediately after depression remission (28). These findings highlight the need for further investigation into the time-course of recovery and how it relates to changes in tree drawing features.

Projective tests, including the Tree Drawing Projection Test, have long been used in psychological assessments, with various elements of the drawings being interpreted as symbolic representations of cognitive, emotional, and unconscious processes. However, these interpretative associations are largely theoretical and remain controversial within the field (5). A significant limitation of projective tests lies in their lack of robust empirical evidence supporting these symbolic associations. While such interpretations have historical and theoretical foundations in psychoanalytic frameworks, contemporary research has not consistently validated the specific meanings attributed to the various components of projective drawings. As a result, the construct validity of these tests remains uncertain.

Furthermore, projective tests have been criticized for their limited reliability and potential for bias in interpretation. Inter-rater reliability, in particular, can be low due to the highly interpretive nature of these tests, which can lead to inconsistencies across different assessors. Without clear, standardized criteria for interpretation, the results of projective tests can vary widely depending on the examiner’s theoretical orientation and personal judgment. In light of these limitations, it is important to acknowledge that while projective tests may offer valuable qualitative insights, their diagnostic utility should be considered with caution. More empirical research is needed to establish a stronger foundation for the symbolic interpretations used in these tests, as well as to develop more reliable and standardized methods for interpreting the drawings.

This study compared the differences in the Tree Drawing Projection Test among the depression group, depression remission group, and normal control group. With further validation, the Tree Drawing Projection Test is expected to become an important tool for assessing the severity of depression and treatment outcomes. However, this study also has several limitations that warrant further exploration in future research. First, although the Tree Drawing Projection Test revealed significant differences between groups, these results demonstrate only group differences rather than diagnostic accuracy. Therefore, the current findings should be interpreted with caution, as this limits their direct application in clinical practice. Future research should further validate the effectiveness of the Tree Drawing Projection Test as a diagnostic tool. Additionally, cultural factors may influence the results of the Tree Drawing Projection Test, especially with regard to emotional expression and psychological projection across different cultural contexts (29–31). As this study was conducted within the Chinese cultural context, the generalizability of the results may be limited. Therefore, we recommend that future studies consider cross-cultural validation to enhance the applicability and generalizability of the Tree Drawing Test across different cultural groups. Finally, although this study provides a new perspective on the quantitative analysis of the Tree Drawing Projection Test, it remains in the preliminary stages of exploration. Future research should refine the methodology and conduct larger-scale validation studies to determine its practical value and potential for clinical diagnostic use.

## Data Availability

The original contributions presented in the study are included in the article/supplementary material. Further inquiries can be directed to the corresponding author.
